# Fluorescent Cellular Assay for Screening Agents Inhibiting *Pseudomonas aeruginosa* Adherence

**DOI:** 10.3390/s150101945

**Published:** 2015-01-16

**Authors:** Libuše Nosková, Božena Kubíčková, Lucie Vašková, Barbora Bláhová, Michaela Wimmerová, Marie Stiborová, Petr Hodek

**Affiliations:** 1 Department of Biochemistry, Faculty of Science, Charles University in Prague, Hlavova 8, 128 40 Prague 2, Czech Republic; E-Mails: noskova.libuse@seznam.cz (L.N.); bojkaku@gmail.com (B.K.); luci.vaskova@gmail.com (L.V.); bara.blahova@volny.cz (B.B.); stiborov@natur.cuni.cz (M.S.); 2 Central European Institute of Technology, Masaryk University, Kamenice 753/5, 625 00 Brno, Czech Republic; E-Mail: michaw@chemi.muni.cz

**Keywords:** cystic fibrosis, lungs, bacterial infection, lectin, chicken antibody, cell line, dual fluorescence

## Abstract

Antibodies against *Pseudomonas aeruginosa* (PA) lectin, PAIIL, which is a virulence factor mediating the bacteria binding to epithelium cells, were prepared in chickens and purified from egg yolks. To examine these antibodies as a prophylactic agent preventing the adhesion of PA we developed a well plate assay based on fluorescently labeled bacteria and immortalized epithelium cell lines derived from normal and cystic fibrosis (CF) human lungs. The antibodies significantly inhibited bacteria adhesion (up to 50%) in both cell lines. In agreement with *in vivo* data, our plate assay showed higher susceptibility of CF cells towards the PA adhesion as compared to normal epithelium. This finding proved the reliability of the developed experimental system.

## 1. Introduction

Cystic fibrosis (CF) is an autosomal recessive disorder caused by mutations in a single gene coding for the CF transmembrane conductance regulator (CFTR) protein [1]. Pathophysiological changes in the lungs of CF patients are responsible for these patients susceptibility toward microbial infections. Frequent and repeated airway bacterial infections with pathogens such as *Pseudomonas aeruginosa* (PA) or *Burkholderia cepacia* complex lead to a chronic endobronchial colonization which ensues in an intense neutrophilic inflammatory response [2]. These conditions lead a to life-threatening lung disease in CF patients [3]. While antibiotics are administered to slow down the decline of the pulmonary function and to reduce the frequency and morbidity of pulmonary exacerbations, their efficiency takes the toll in the development of bacteria resistance [4]. This is why there is an urgent need to develop novel and effective ways of therapy (for review see [5]). In addition to efforts in the area of CF gene therapy and corrections of CFTR function, the antimicrobial management—such as CF patient immunization against invading pathogens—is being extensively studied [6]. However, the concept of immunization of CF patients with vaccines derived from PA virulence factors suffers from two shortcomings: (I) the raised anti-pseudomonal immunoglobulins bind PA and therefore induce lung epithelium inflammatory damage; and (II) in general the secretion of immunoglobulins on CF mucosal membranes is impaired [3]. Thus, the passive immunization via non-inflammatory anti-pseudomonal immunoglobulins seems to be a feasible way of preventing PA lung infection [7]. In this respect, chicken yolk antibodies (IgY) provide a great potential in becoming an efficient tool of passive immunization [8]. The most significant advantage of IgY, in contrast to mammalian IgG, consists in their inability to induce inflammatory reaction when binding the antigen. Moreover, the large production of IgY (100 mg/yolk) makes these antibodies well suited for prophylaxis of bacterial infections [9]. Our previous experiments carried out with rats have shown that inhalation of nebulized IgY induced no lung pathology in experimental animals [10]. Because the bacteria adherence to epithelial cells serves as an important initial step in the onset of PA infection, the prophylactic IgY might inhibit this process. In case of CF patients, their airway surfaces lack the sialylation of glycoconjugates such as GM1 [11–13]. That facilitates PA binding and thus increases susceptibility of lungs to PA colonization [14]. Thus, in this study we developed an experimental set-up examining the effect of various compounds on bacteria adhesion to epithelial cells. Since the PA lectin, PAIIL, is considered to be involved in bacteria adhesion on CF airway cells [15], we prepared chicken yolk antibodies against recombinant PAIIL and tested them in this system.

## 2. Experimental Section

### 2.1. Antibody Preparation

Antibodies were prepared from egg yolks laid by chickens immunized with recombinant PA lectin, PAIIL, as described elsewhere [9,12]. Pre-immune IgY sample (control) was purified from eggs collected a week prior to the immunization. The presence of anti-PAIIL IgY was determined on ELISA and Western blots using PAIIL and PA lysate as antigens, respectively. The antibody titer was estimated to be 5 μg/mL.

### 2.2. Cell Staining

Cells were stained with fluorescent PKH dyes (Sigma, St. Louis, MO, USA) according to the manufacturer's protocol. Briefly, harvested epithelial cells NuLi or CuFi (immortalized epithelium cell lines derived from normal or CF human lungs, respectively, purchased from ATCC) were washed with PBS, resuspended in Diluent C and incubated for 5 min with an equivalent volume of 4 μM PKH67 (in Diluent C). Upon that, the staining process was stopped with the addition of FBS (2-fold volume excess) and cells were washed repeatedly with BEGM by centrifugation (1000× *g* for 5 min) to remove an excess of the dye. Patient isolate (# ST1763) of *P. aeruginosa* was grown in suspension culture either in minimal mineral medium “M9” (with 0.2% glucose) or in rich medium PS (peptone/casein digest). Bacterial cells were fluorescently labeled with PKH26 as follows: cells at an exponential growth phase were collected, washed with PBS and resuspended in Diluent C to make 6 × 10^8^ CFU/mL. Bacterial suspension was mixed (1:1) with 20 μM PKH26 (in Diluent C) and incubated for 30 min. To terminate the staining, 2 fold excess of 1% BSA in PBS was added and cells were extensively washed with PBS by repeated centrifugation (11,000× *g* for 10 min) to remove excess of the dye.

### 2.3. Bacterial Adhesion Assay

NuLi or CuFi cells stained with a fluorescent dye PKH67 were seeded (5 × 10^5^ cells/well) onto well plates (24 wells) and incubated for 24 h at 37 °C, 5% CO_2_ to form a confluent layer. Bacteria *P. aeruginosa* labeled with PKH26 was pre-incubated for 10 min with antibodies, anti-PAIIL or pre-immune IgY (1 mg/mL), saccharides, L-fucose or D-galactose (1% solution) or PBS and then applied (300 μL) onto well plates. The input ratio was about 30 bacteria per epithelial cell. After incubation at room temperature (up to 2 h) non-adhered bacteria were removed by extensive washing with PBS. The adhered PA on epithelial cells were quantified fluorometrically using spectrofluorometer Tecan Infinite M200 Pro (excitation (Ex) at 522 nm, emission (Em) at 569 nm). Simultaneously, the fluorescence of epithelial cells was determined (Ex 470 nm, Em 505 nm). Results were expressed as a relative fluorescence ratio PA/NuLi or PA/CuFi. Experimental data were statistically analyzed by SigmaPlot 2000 graphing and statistical analysis software.

## 3. Results and Discussion

**Figure 1.**
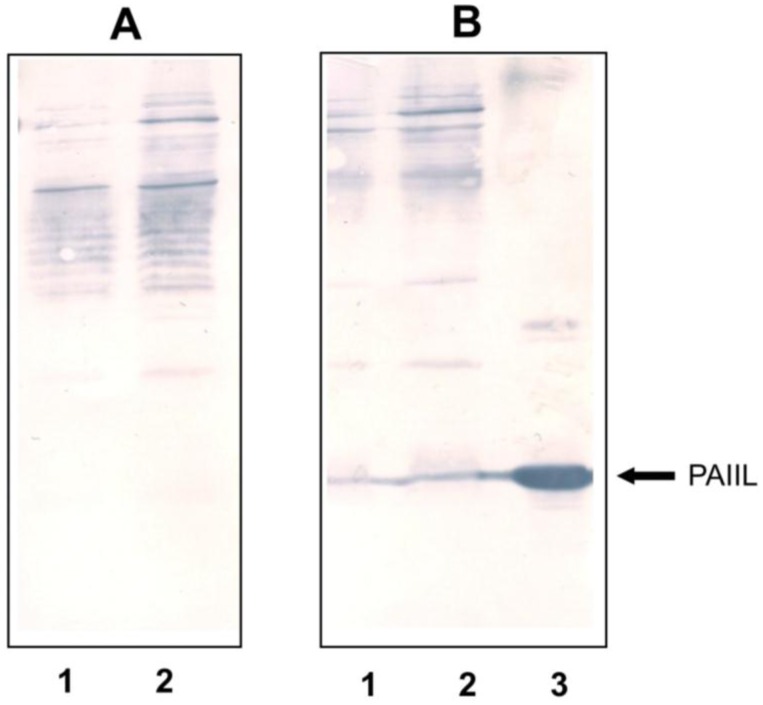
Western blot analysis of *Pseudomonas aeruginosa* lectin (PAIIL). Samples containing PAIIL, cell lysate of PA grown in rich medium PS (line **1**) or in minimal mineral medium M9 (line **2**) and recombinantly expressed PAIIL (line **3**) were separated on reduced 10% SDS PAGE. Separated proteins were electrotransfered onto PVDF membranes and developed with pre-immune IgY (panel **A**) and anti-PAIIL IgY (panel **B**) at 30 μg/mL concentrations. The arrow marks the position of PAIIL protein band.

In order to develop a tool for PA prophylaxis preventing bacteria adherence to CF airway epithelial cells, we prepared chicken yolk antibodies against PAIIL. This bacterial lectin is assumed to enhance the PA binding to CF cell glycoconjugates with impaired sialylation [11,15]. ELISA of IgY fractions of yolks confirmed that specific anti-PAIIL IgY was effectively produced in the course of the immunization procedure (not shown). Anti-PAIIL IgYs were further examined on Western blots to prove their binding activity with PAIIL in bacteria lysates. As shown in Figure 1, anti-PAIIL IgY recognized PAIIL both in recombinant sample and in PA lysate. Blots developed with pre-immune IgY showed no reactivity with PAIIL (panel A), thus they served in further experiments as a control IgY. The cultivation of PA in mineral medium “M9” [16] did not significantly induce the production of PAIIL as compared with common medium PS (see Figure 1). This discrepancy might be attributed to the variability of PA strains, as PA used in our experiments was a clinical isolate ST1763 but not a strain 1244-NP.

**Figure 2.**
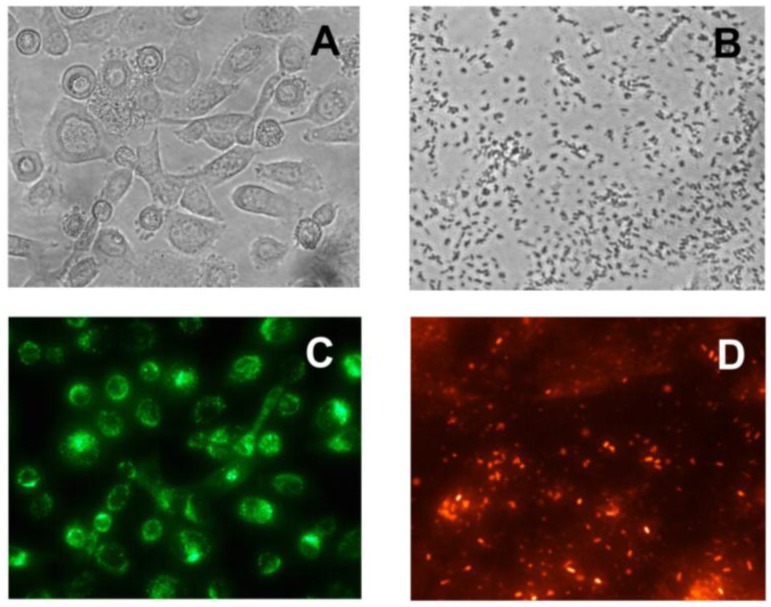
Micrographs of epithelium cells (**A**, unstained; **C**, stained with PKH67) and *Pseudomonas aeruginosa* (**B**, unstained; **D**, stained with PKH26). Fluorescent cells were examined on a Nikon Eclipse microscope equipped with a filter 31001 FITC C87701 for epithelium cells (PKH67) and filter 31002 RdiI C87702 for PA (PKH26).

**Figure 3.**
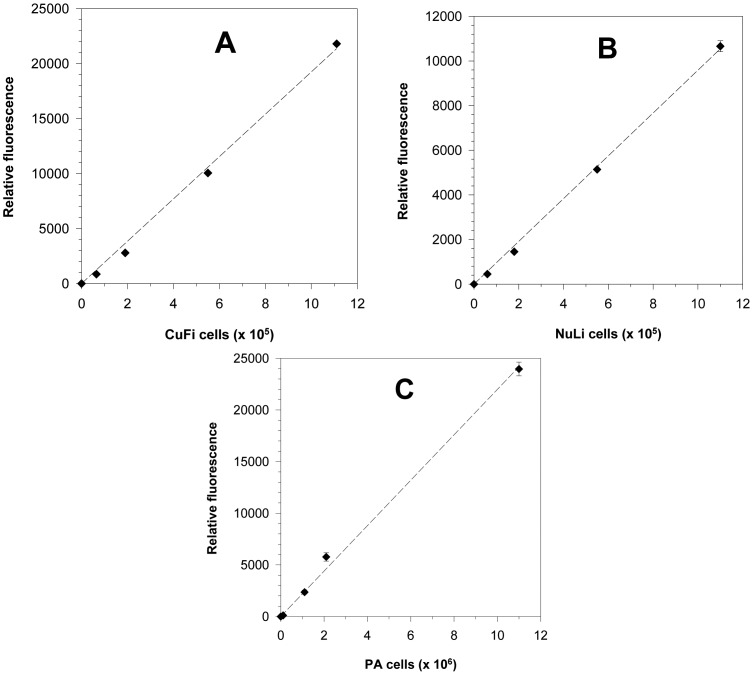
Calibration plots of PKH stained cells. Relative fluorescence of cells was determined on a spectrofluorometer (Tecan Infinite M200 Pro) set for PKH67 (Ex 522 nm, Em 569 nm) and for PKH26 (Ex 470 nm, Em 505 nm). Epithelium cells CuFi and Nuli are shown in plots **A** and **B**, respectively. Plot **C** depicts the calibration line for fluorescently labeled PA. Relative fluorescence of background (blank) was subtracted from that of cell samples. The values are means ± SD of five independent determinations.

To evaluate anti-PAIIL IgY as a potential tool for PA prophylaxis of CF patients, an *ex vivo* bacterial adhesion assay was developed. The experimental set-up is based on the use of airway epithelium cells adhered on well plates, which were exposed to PA in the presence or absence of anti-PAIIL IgY. The epithelial cell line derived from CF-patient lung tissues (CuFi) was employed to mimic conditions of CF subjects. Cells from airways of a healthy donor (NuLi) were included in the assay for comparison. The precise quantification of adhered PA on epithelial cell monolayer was made possible by labeling the bacteria and epithelial cells with fluorescent dyes. Lipophilic cell tracking PKH dyes are well suited for the assay design as they do not affect cell surface structures important for PA adhesion, show a low impact on the cell viability, and allow a long-term monitoring of cells *in vivo*. Moreover, the fluorescence of PKH26 can be measured without interference with PKH67 dye that is essential for the assay set-up when both cells are simultaneously present in the well. Figure 2 represents micrographs of PA and epithelial cells stained with red fluorescent PKH26 and green fluorescent PKH67 dyes, respectively. Stained CuFi, NuLi and PA cells showed a linear relationship between the number of cells and their relative fluorescence (see Figure 3) which makes the cell quantification possible.

**Figure 4.**
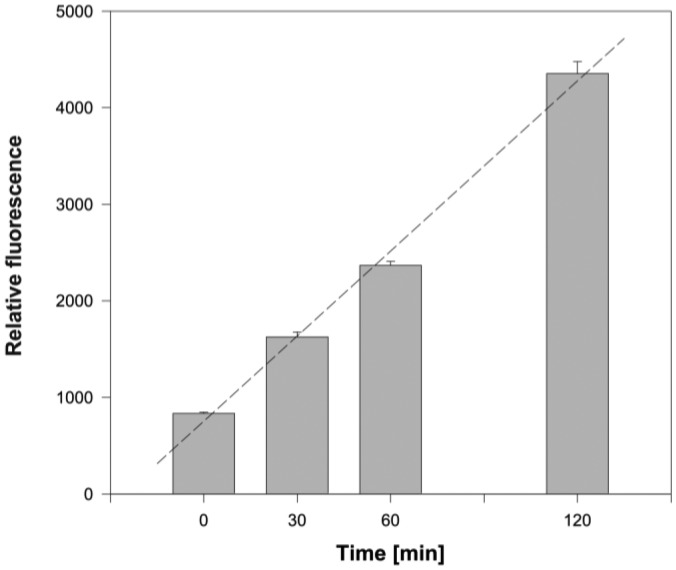
Time course of the PA adherence on an epithelial cell monolayer. Fluorescently labeled PA bacteria (1.5 × 10^7^ CFU) were applied to wells and incubated at room temperature. At distinct time points bacteria were removed and the wells washed with PBS. Adhered PA bacteria were quantified using spectrofluorometer (Tecan Infinite M200 Pro) set for PKH26 (Ex 470 nm, Em 505 nm). Plotted data are means ± SD of four independent incubations.

In pilot experiments the length of PA incubation with monolayers of epithelial cells was examined. After the removal of non-adherent bacteria and extensive washing of wells, the number of *P. aeruginosa* associated with epithelial cells was determined. Under the input ratio of about 30 bacteria per epithelial cell the number of bound PA cells increased almost linearly with time during the course of incubation (see Figure 4). The PA adhesion seems to be rather fast as even the momentary exposure of epithelial cells to PA followed by their immediate removal (time 0 in Figure 4) resulted in detectable amounts of retained PA. To gain a readout of relative fluorescence high enough for precise quantification of adhered PA, the 2-h incubation was chosen for further adhesion experiments. These conditions were used to test the efficacy of anti-PAIIL IgY in the PA adhesion assay with both cell lines. Results are expressed as relative fluorescences of PA normalized per number of NuLi/CuFi cells. This arrangement eliminates possible variations caused by differences in the number of cells in monolayers.

**Figure 5.**
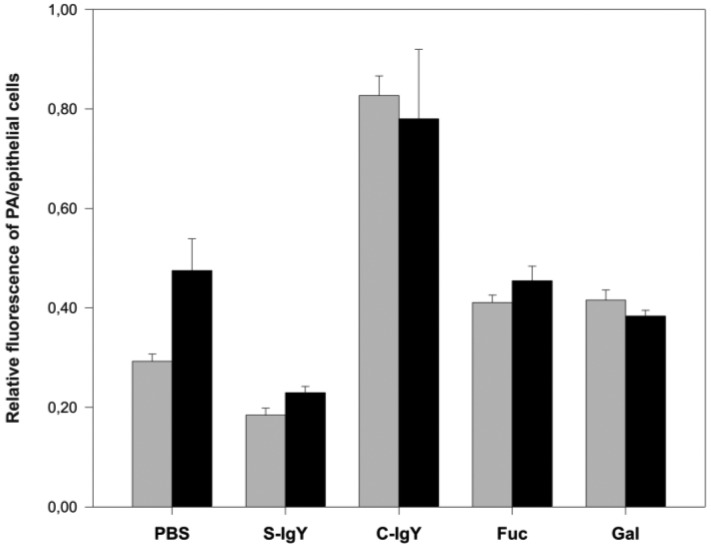
Adhesion of PA to epithelium cells in the presence of IgYs and saccharides. Monolayers of NuLi (grey bars) and CuFi (black bars) were exposed to PA suspension containing anti-PAIIL IgY (**S-IgY**), pre-immune IgY (**C-IgY**), L-fucose (**Fuc**), D-galactose (**Gal**) or PBS as a control. After a 2-hour incubation non-adhered bacteria were discarded and retained PA and epithelial cells were quantified using spectrofluorometer (Tecan Infinite M200 Pro). Results are expressed as a relative fluorescence ratio of PA/NuLi or PA/CuFi. Plotted data are means ± SD of four independent incubations.

As shown in Figure 5, the presence of anti-PAIIL IgY during the PA incubation significantly lowered the number of bacteria associated with either CuFi or NuLi cells (compared to PA treated with PBS only). More profound protecting effect of anti-PAIIL IgY against PA binding (>50% of PBS control) was observed for CuFi as compared with NuLi cells (∼37% of PBS control). Surprisingly, pre-immune IgY controls (C-IgY, see Figure 5) stimulated the PA association with CuFi and NuLi monolayers. This unexpected effect might be attributed to the agglutination of PA via highly mannosylated glycoconjugates present on each heavy chain of IgY which bind to lectin PAIIL. Resulting PA aggregates (observed by fluorescent microscope) retain PA in larger numbers in comparison with single bacteria after PBS washings of wells. In the case of anti-PAIIL IgY, the saccharide binding site of PAIIL lectin is blocked by the specific immunoglobulin and thus excluded from the interaction with saccharides of IgY heavy chain. This finding documents not only the ability of anti-PAIIL IgY to bind PAIIL on the *P. aeruginosa* surface but also to inhibit PAIIL saccharide binding activity. It is important to note that CF cells (CuFi, see Figure 5) are more susceptible (∼30%) to PA adhesion than normal epithelium (NuLi). These data show that the developed *ex vivo* assay system approximates well *in vivo* conditions of CF patients. Moreover, our results provide the evidence that to some extent PAIIL is responsible for *P. aeruginosa* colonization of CF patients.

In fact, the PA adhesion is facilitated by two lectins, PAIL and PAIIL, which bind to saccharides on the surface of airway epithelial cells [15]. Thus, PA lung infection might be also prevented by D-galactose and L-fucose, which compete for the binding sites on PAIL and PAIIL, respectively. While the inhalation of these saccharides reduced the *P. aeruginosa* counts in CF patients [17], in our cellular assay D-galactose and L-fucose failed in the protection of epithelial cells against PA adhesion. In case of CuFi cells, some marginal reduction of PA binding was observed for both saccharides, however, with NuLi monolayers the presence of saccharides significantly increased the bacteria adherence (see Figure 5). This observation might be explained *via in vivo* experiments with mice showing that unmodified sugars, contrary to *i.e.*, Me-α-Gal for PAIL and Me-α-Fuc for PAIIL, inhibit lectin to a lower degree [13]. Although the saccharides were apparently not effective in the inhibition of PA binding, the lower values for CuFi monolayers (compared to NuLi) suggest some competition of applied saccharides and non-sialylated glycoconjugates of CF epithelial cells for the PA lectin binding.

## 4. Conclusions

The new cellular assay system based on dual fluorescence determination was developed and used to study *P. aeruginosa* adherence inhibition. Herein we report for the first time that chicken yolk antibodies developed against PAIIL are effective in reducing bacteria adhesion on human airway epithelia cells. Our results suggest that the anti-PAIIL IgY may provide an alternative therapeutical approach in prevention of *P. aeruginosa* infections. The proven anti-PAIIL efficacy of IgYs allows us to extend our research further and examine prophylactic properties of these antibody on animals experimentally infected with PA.
